# Meat Food Group Intakes and the Risk of Type 2 Diabetes Incidence

**DOI:** 10.3389/fnut.2022.891111

**Published:** 2022-06-30

**Authors:** Firoozeh Hosseini-Esfahani, Niloofar Beheshti, Glareh Koochakpoor, Parvin Mirmiran, Fereidoun Azizi

**Affiliations:** ^1^Nutrition and Endocrine Research Center, Research Institute for Endocrine Sciences, Shahid Beheshti University of Medical Sciences, Tehran, Iran; ^2^Maraghe University of Medical Sciences, Maragheh, Iran; ^3^Endocrine Research Center, Research Institute for Endocrine Sciences, Shahid Beheshti University of Medical Sciences, Tehran, Iran

**Keywords:** type 2 diabetes, protein, food groups, legumes, red meat, nut, fish, poultry

## Abstract

**Aim:**

This study aimed to evaluate the association of meats and their substitute food group intakes, including nuts, eggs, and legumes, with type 2 diabetes (T2D).

**Methods:**

For this secondary analysis, we selected eligible adults (*n* = 6,112) from the Tehran Lipid and Glucose Study participants with a median follow-up of 6.63 years. Expert nutritionists assessed dietary intakes using a valid and reliable semiquantitative food frequency questionnaire. Biochemical and anthropometric variables were assessed at baseline and follow-up examinations. We used multivariable Cox proportional hazard regression models to estimate the new onset of T2D concerning meats and their substitute food groups.

**Results:**

We performed this study on 2,749 men and 3,363 women, aged 41.4 ± 14.2 and 39.1 ± 13.1 years, respectively. The number of participants with incident T2D was 549. After adjusting for confounders, legume [HR: 1, 0.74 (0.58–0.94), 0.69 (0.54–0.90), 0.65 (0.50–0.84), *P*-trend = 0.01)] was inversely associated with incident T2D. Fish intake [HR: 1, 1.0 (0.79–1.27), 1.17 (0.91–1.50), 1.14 (0.89–1.45), *P*-trend = 0.01)] was positively associated with incident T2D. In subjects who reported poultry consumption of 36.4–72.8 g/day, a positive association [HR: 1.33 (1.03–1.71)] between poultry intake and T2D risk was observed.

**Conclusion:**

Our findings revealed that a diet rich in legumes significantly reduced the risk of T2D incidence, while a diet high in poultry increased the risk of T2D incidence, probably due to high-temperature cooking methods and environmental contaminants.

## Introduction

The prevalence of type 2 diabetes (T2D) mellitus is increasing rapidly due to changes in lifestyle such as alteration of diet into unhealthy eating behaviors (1). Researchers have recognized effective food and dietary factors, which play essential roles in preventing diabetes. A systematic review and meta-analysis of prospective studies showed that the increasing consumption of whole grains, vegetables, fruits, and dairy and decreasing consumption of red and processed meat and egg intakes could cause a significant change in the risk of T2D (2).

Many studies investigated the relationship of protein consumption with diabetes risk, but the results are still controversial (3, 4). In this regard, several studies claimed that red and processed meat could increase the risk of T2D (5–7). In comparison, other studies found that diets rich in plant-based protein food could lower the risk of T2D (8, 9). Many investigations have reported the role of impaired glucose and insulin metabolism in the development of T2D, and different protein sources could affect them in disparate ways (10, 11). In contrast, the quantity of meat consumption also plays a substantial part in the formation of T2D (4, 9); one meta-analysis showed that the consumption of 100 g red meat/day and 50 g processed meat/day increased the risk of T2D for 19 and 51%, respectively (7). This association remains a question regarding low red and white meat consumption among the Iranian population (12).

Despite extensive studies in this field, studies have not yet been able to find an accurate answer on the role of red and white meat and its substitutes for the development of T2D. As far as we know, there is no study about the association between red and white meat and its substitutes for T2D in the Middle East. This study aimed to evaluate the association between the total intake of meats and their substitutes, including nuts, eggs, and legumes, with the risk of T2D incidence in a group of Tehranian adults.

## Materials and Methods

### Study Population

We selected participants from the Tehran lipid and glucose study (TLGS), a large-scale population-based cohort study conducted to determine risk factors for non-communicable diseases in a representative sample of Tehran, the capital of Iran (13, 14). The first examination survey was operated from 1999 to 2001 on 15,005 individuals aged? ≥?3 years, using the composite stratified cluster random sampling technique. The cohort study supervised follow-up examinations every 3 years; 2002–2005 (survey 2), 2005–2008 (survey 3), 2008–2011 (survey 4), 2012–2015 (survey 5), and 2015–2018 (survey 6) to identify recently developed diseases.

**FIGURE 1:**
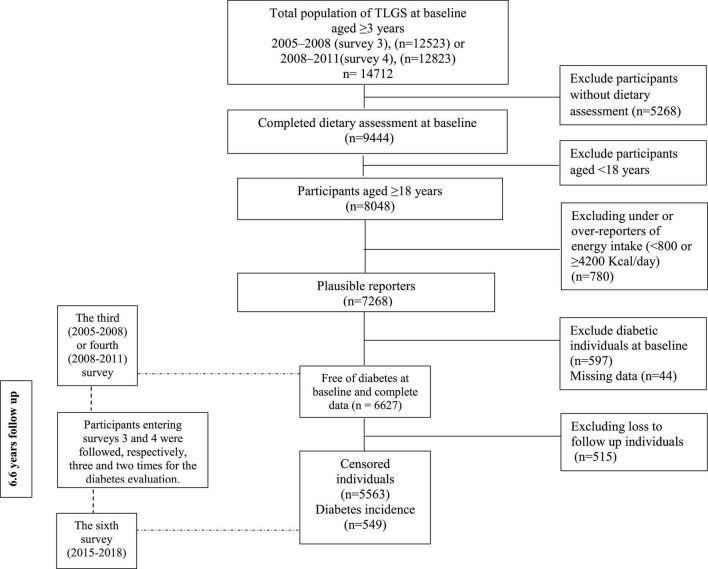
Outline of study participants’ selection.

Of individuals participating in surveys 3 and 4, we randomly selected 8,048 subjects aged ≥ 18 years that completed the dietary assessment for this secondary analysis. After excluding subjects with underreporting or overreporting of energy intake (<800 or ≥ 4,200 kcal/day) (15) (*n* = 780), we selected a total of 7,268 adult men and women with available dietary, biochemical, and anthropometric data as the baseline population and followed until survey 6 (participants entering at surveys 3 and 4 were followed, respectively, three times and two for the outcome measurements). Of these participants, we excluded pregnant or lactating women and subjects diagnosed with T2D based on measurements of fasting plasma glucose at baseline or self-reported taking glucose-lowering drugs (16) (*n* = 597). Finally, after excluding participants missing any follow-up data (*n* = 515), 6,112 subjects remained and entered the analysis (Figure 1). The sample size was calculated based on α (two-tailed): 0.05, β: 0.2, q1: 0.3, q0:0.7, and relative hazard (RH): 1.25. We determined the effect size for estimating sample size based on previous literature on the relationship between red and processed meat, poultry, fish, eggs, and legumes with diabetes, which was in the range of 1.0–1.51, so the effect size of 1.25 was estimated as a middle of this range (7, 8, 17–20).

All participants signed a written informed consent before taking part in this investigation. The ethics committee of the Research Institute for Endocrine Sciences, Shahid Beheshti University of Medical Sciences (Tehran, Iran) accepted the study protocol.

### Dietary Intake Measurements

Expert nutritionists accomplished dietary assessment using a valid and reliable 168-item semiquantitative food frequency questionnaire (FFQ). They collected usual dietary intakes during the last year through face-to-face personal interviews based on standard portion sizes. In the previous study, nutritional data were collected monthly using twelve 24 h dietary recalls and two 168-item semiquantitative FFQs to determine the relative validity and reliability of the FFQ (21, 22). The mean energy-adjusted correlation coefficients for overall nutrient intakes between the 24 h dietary recalls and FFQ2 were 0.44 and 0.37 in ≤ 35-year-olds and > 35-year-olds, respectively (22).

The consumption frequency of each food item on a daily, weekly, monthly, or yearly basis was converted to daily intakes; portion sizes were then converted to mass (g/day) using household measures. Due to the incompletely Iranian food composition table (FCT), the United States Department of Agriculture (USDA) FCT was used to analyze the nutrient composition of food items (e.g., bread, legume, nuts, white, or red meat) not included in the Iranian FCT.

Meat food groups and their plant substitutes were defined as the sum of plant or animal sources. Plant substitute of meat food group was defined as the sum of nuts, legumes, and soy (g/day), and animal sources include beef, lamb, organ meats (kidney, beef liver, and heart), processed meats (sausages and hamburger), eggs, fish, and poultry (g/day) (23).

### Physical Activity Measurements

An interviewer measured physical activity levels using a Persian-translated modifiable activity questionnaire (MAQ). The previous study reported high reliability and moderate validity of this questionnaire (24). Time and frequency of light, middle, high, and challenging intensity activities were received as stated by routine activities of daily life over the past year. These activity data were converted into metabolic equivalent/minutes/week (MET/min/week) (25).

### Blood Pressure and Anthropometric Measurements

Trained staff measured body weight using a digital scale (Seca 707) with an accuracy of 100 g in conditions where people had minimum clothes and were barefoot. A tape measure with an accuracy of 0.5 cm was applied to estimate height in the standing position without shoes and straightened shoulders. Trained staff measured the waist circumference (WC) in the umbilical region after a normal exhalation without pressure on the body surface in the standing position and with the most miniature clothing. They documented measurements to the nearest 0.1 cm.

Using a standard mercury sphygmomanometer, qualified physicians evaluated systolic and diastolic blood pressure (SBP and DBP). They asked participants to rest for 15 min, and then the physician measured blood pressure in the sitting position while setting the cuff on the right arm. They repeated this operation twice with an interval of 30 s. We took the average of two measures in the analysis.

### Biochemical Analysis

Blood samples were collected between 7:00 and 9:00 a.m., after 12–14 h of overnight fasting. The samples were centrifuged 30–45 min after collection. The technician analyzes blood samples using the Selectra 2 auto-analyzer; they carried out all biochemical analyses at the TLGS research laboratory on the day of blood collection. They quantified fasting blood glucose (FBG) concentration using the enzymatic colorimetric method and glucose oxidase technique (Vital Scientific, Spankeren, the Netherlands). Individuals on glucose-lowering drugs were excluded from the study at the study’s baseline. In follow-up times, they performed the standard 2-h post-challenge blood glucose test using oral administration of 82.5 g glucose monohydrate solution (equivalent to 75 g anhydrous glucose) for all individuals who were not on glucose-lowering drugs.

High-density lipoprotein cholesterol (HDL-C) concentration was evaluated after precipitation of the apolipoprotein B-containing lipoproteins with phosphotungstic acid. Triglyceride (TG) level was assessed by enzymatic colorimetric tests using glycerol phosphate oxidase (Pars Azmoon Inc., Tehran, Iran). For glucose, the inter- and intra-assay coefficients of variations were both 2.2%. For TG, inter- and intra-assay coefficients of variations were 1.6 and 0.6%, respectively (26).

### Diabetes Risk Score

The diabetes risk score (DRS) was measured as follows to reduce the number of confounding factors in the analysis: family history of T2D (5 points) (a positive family history of diabetes was determined as having at least one parent or sibling with diabetes), SBP (mmHg) < 120 (0 points), 120–140 (3 points), SBP ≥ 140 (7 points); TG/HDL-C: < 3.5 (0 points), ≥ 3.5 (3 points); waist-to-height ratio: < 0.54 (0 points), 0.54–0.59 (6 points), ≥ 0.59 (11 points); FBG (mmol/L): < 5.0 (0 points), 5.0–5.5 (12 points), 5.6–6.9 (33 points) (27).

### Outcome Definition

Incidence of T2D was determined as fasting plasma glucose concentrations ≥ 126 mg/dl or 2-h plasma glucose concentrations ≥ 200 mg/dl or self-reported taking glucose-lowering drugs (oral diabetes medication or insulin injections) (16).

### Statistical Analysis

We performed statistical analyses using the Statistical Package for Social Sciences (version 21.0; SPSS). A two-sided *P*-value < 0.05 was considered statistically significant. We categorized data into quartiles of plant and animal sources of meat food groups. Chi-square test for categorical variables and one-way ANOVA for continuous variables were used to compare the mean and frequency of participants’ baseline characteristics across quartiles of plant and animal sources of meat food groups. *P* for trend across plant and animal sources of meat food group intake categories were performed by assigning continuous variables in a linear regression model. The normality of variables was checked. Interquartile ranges were written for non-normal distribution variables. The Kruskal-Wallis test was applied to examine the difference across quartiles of animal and plant sources of meat food groups. Multivariable Cox proportional hazard regression analyses were performed to estimate the hazard ratio (HR) and 95% confidence interval (CI) of incident T2D. There were no interaction terms of total meat food groups with age or sex. The first quartile was assumed as the reference. The median of each quartile was utilized as a continuous variable to estimate the *P*-value of trends across quartiles of total animal and plant sources of meat food groups in the Cox proportional hazard regression models. The confounders were selected based on literature; also, we applied each confounder in the univariable Cox regression model; a two-tailed *P*-value < 0.20 was practiced for specifying admission in the model.

Time to event was defined as the time between baseline and the event date (for event cases) or the last follow-up (for censored participants), whichever occurred first. The event date was defined as the mid-time between the follow-up visit date at which T2D was detected for the first time and the most recent follow-up visit before the diagnosis. Study participants were censored due to death, loss to follow-up, or non-occurrence of T2D before the end of follow-up. The Cox regression models were adjusted for several potential confounders. The analyses were performed without adjustment (model 1), and model 2 was adjusted for sex and age. In model 3, education levels (> 14 and ≤ 14 years), smoking (never smoked, past smoked, and current smoker), body mass index (BMI), physical activity, diabetes risk score, total energy, fat, and fiber intakes (continuous) were added to model 2. Non-normal variables were transformed into Ln variables to have a normal distribution and then entered into the models. The proportional hazard assumption was verified using the Schoenfeld residuals test and plot of log [-log (survival)] vs. log (time) to see if they are parallel.

## Results

Our study was performed on 2,749 men and 3,363 women (*n* = 6,112), respectively, aged 41.4 ± 14.2 and 39.1 ± 13.1 years, including 5,563 censors and 549 incident cases of T2D during a mean of 6.63 years of follow-up. The distribution of baseline characteristics of the participants across quartiles of animal and plant sources of meat food group intakes is shown in Table 1. This study revealed that those who consumed higher animal sources of meat food groups were younger. The level of physical activity was higher in the upper quartiles of animal sources of meat food group intakes than lower quartiles. The percentage of current smokers was higher in the upper quartiles of animal sources of meat food groups than lower quartiles.

**TABLE 1:** Baseline characteristics of adult participants of the Tehran lipid and glucose study across quartiles of animal and plant sources of meat food groups.

	Animal sources of meat food groups				*P* *	Plant substitute of meat food groups				*P* *
	Q1	Q2	Q3	Q4		Q1	Q2	Q3	Q4	
Min-max	0-51.90	51.91–75.67	75.69-110.09	110.1–319.9		0–15.61	15.62–28.61	28.66–52.08	52.11–192.18	
Total meat food groups (gr/day)	68.3 ± 31.8	101 ± 34.8	133 ± 43.5	209 ± 78	<0.001	86.5 ± 53.1	107 ± 55	132 ± 58.3	188 ± 78.9	<0.001
Total meat food groups/body weight	0.99 ± 0.52	1.46 ± 0.57	1.90 ± 0.73	1.19 ± 0.03	<0.001	1.24 ± 0.77	1.51 ± 0.81	1.86 ± 0.84	2.66 ± 1.19	<0.001
Plant sources of meat food groups (gr/day)	11.0–39.1*	14.7–49.1	17.8–53.7	19.5–62.0	<0.001	6.07–12.1	18.5–24.7	32.9–43.9	62.3–96.8	<0.001
Plant sources of meat food groups/body weight	0.16–0.57*	0.21–0.70	0.23–0.76	0.26–0.84	<0.001	0.08–0.17	0.24–0.36	0.44–0.63	0.85–1.42	<0.001
Animal sources of meat food groups (gr/day)	37.6 ± 10.6	63.8 ± 6.87	91.2 ± 9.81	160 ± 51.8	<0.001	77.0 ± 49.9	84.6 ± 50.2	92.5 ± 53.0	98.9 ± 56.6	<0.001
Animal sources of meat food groups/body weight	0.55 ± 0.19	0.92 ± 0.20	1.30 ± 0.30	2.21 ± 0.82	<0.001	1.11 ± 0.73	1.19 ± 0.73	1.31 ± 0.76	1.39 ± 0.81	<0.001
Age (years)	44.1 ± 14.4	40.5 ± 13.5	38.6 ± 12.8	37.2 ± 12.8	<0.001	39.8 ± 14.1	39.6 ± 13.2	39.7 ± 13.6	41.4 ± 13.5	<0.001
Women (%)	61.7	59.7	54.2	44.7	<0.001	57.7	56.3	55.5	50.0	<0.001
Current smokers (%)	17.3	18.6	23.5	29.9	<0.001	24.0	22.4	22.7	20.3	0.07
Physical activity (MET/min/week)	80.4–615*	89.3–714	96.9–720	93.7–816	<0.001	89.3–658	89.3–658	89.3–714	89.3–763	0.33
Education level ≥ 14 years (%)	23.2	28	29.5	28.4	<0.001	25.8	28.8	30.7	29.3	0.03
BMI (Kg/m2)	27.2 ± 4.9	27.0 ± 4.6	26.9 ± 4.8	26.9 ± 4.7	0.27	26.7 ± 4.9	27.3 ± 4.8	27.0 ± 4.6	26.9 ± 4.6	0.03
Waist circumference (cm)	89 ± 13.4	88.3 ± 12.7	88.9 ± 12.7	89.7 ± 13.9	0.34	88.6 ± 13.3	89.4 ± 13.1	89.3 ± 13.3	89.1 ± 12.6	0.30
SBP (mmHg)	113 ± 17.2	111 ± 15.2	111 ± 15.8	111 ± 15.8	0.004	111 ± 16.4	111 ± 15.7	112 ± 15.5	113 ± 16.5	<0.001
DBP (mmHg)	74.6 ± 10.8	74 ± 10.6	74.4 ± 10.5	75.0 ± 10.8	0.06	73.7 ± 10.7	73.9 ± 10.5	74.8 ± 10.9	75.5 ± 10.6	<0.001
Total cholesterol (mg/dL)	138 ± 63.0	184 ± 67.8	231 ± 80.1	335 ± 185	<0.001	195 ± 136	217 ± 119	232 ± 133	246 ± 136	<0.001
FBG (mg/dL)	90.8 ± 9.47	90.2 ± 8.9	90.4 ± 8.9	90.6 ± 9.1	0.25	88.2 ± 9	90.5 ± 9.2	91.1 ± 8.7	92.3 ± 9	<0.001
Family history of diabetes (%)	13.7	13.7	13.3	14.3	0.68	17.1	14.3	13.1	10.3	<0.001
Energy intake (kcal/day)	1,909 ± 608	2,170 ± 587	2,379 ± 611	2,726 ± 632	<0.001	1,988 ± 635	2,188 ± 649	2,385 ± 629	2,642 ± 624	<0.001
Carbohydrate (% of energy)	60.2 ± 7.5	58.7 ± 6.5	58.0 ± 6.6	56.3 ± 10.8	<0.001	58.2 ± 7.6	58.2 ± 6.9	58.3 ± 6.7	58.5 ± 10.8	0.77
Protein (% of energy)	13.4 ± 4.1	13.9 ± 2.4	14.4 ± 2.6	15.9 ± 10.2	<0.001	13.7 ± 2.6	14.2 ± 3.5	14.5 ± 3.5	15.3 ± 10.3	<0.001
Total fat (% of energy)	29.8 ± 7.3	30.5 ± 6.5	30.7 ± 6.6	31.5 ± 22.9	0.003	30.6 ± 7.2	30.7 ± 6.8	30.5 ± 6.3	30.8 ± 23.2	0.95
SFA (% of energy)	9.8 ± 3.1	10.0 ± 2.8	10.2 ± 5.5	10.8 ± 22.9	0.15	10.4 ± 3.1	10.2 ± 2.9	10.1 ± 5.6	10.2 ± 23.1	0.84
MUFA (% of energy)	10 ± 3.2	10.3 ± 2.8	10.4 ± 2.7	11.2 ± 22.8	0.04	10.5 ± 2.9	10.5 ± 3	10.3 ± 2.7	10.5 ± 23.1	0.9
PUFA (% of energy)	6.18 ± 2.4	6.25 ± 2.12	6.25 ± 2.15	6.74 ± 22.9	0.51	6.1 ± 2.3	6.1 ± 2.2	6.1 ± 1.9	6.8 ± 23.1	0.27
Sugar-sweetened soft drinks (ml/day)	0.76–18.6*	1.91–28.5	3.96–40.0	6.66–60.0	<0.001	1.53–28.5	2.30–28.5	2.73–40.0	1.64–28.5	0.006
Fiber (gr/1,000 Kcal)	9.43 ± 3.49	9.44 ± 3.16	9.53 ± 3.23	9.08 ± 3.68	0.001	8.01 ± 3.13	8.90 ± 2.91	9.61 ± 2.92	11.2 ± 3.71	<0.001

*Values are mean ± SD unless otherwise listed, ANOVA test; P for trend, across animal and plant sources of meat food group categories were performed by assigning continuous variables in a linear regression model, and chi-square test was applied for categorical variables.*

*Interquartile range for non-normal distribution, Kruskal-Wallis test.

*Q, quartiles of animal sources of meat food group and plant substitute of meat food group consumption; MET, metabolic equivalent; BMI, body mass index; MUFA, monounsaturated fatty acids; PUFA, polyunsaturated fatty acids; SFA, saturated fat; SBP, systolic blood pressure; DBP, diastolic blood pressure, FBG, fasting blood glucose.*

Furthermore, the numbers of highly educated people in both animal and plant sources of meat food groups were higher in the upper quartiles than in the lower quartiles.

Moreover, subjects in the upper quartiles of animal sources of meat food group intakes had higher intakes of total energy, protein, total fat, monounsaturated fatty acid (MUFA), and sugar-sweetened beverages compared with the lower quartiles. Subjects who consumed higher plant sources of meat food groups had higher age than lower intakes. The levels of SBP, DBP, total cholesterol, and FBG of participants in higher quartiles of plant sources of meat food groups were higher than those in lower quartiles. Furthermore, individuals with higher intakes of plant substitutes of meat food groups were higher energy, protein, sugar-sweetened soft drinks, and fiber intakes than those in lower quartiles.

**FIGURE 2:**
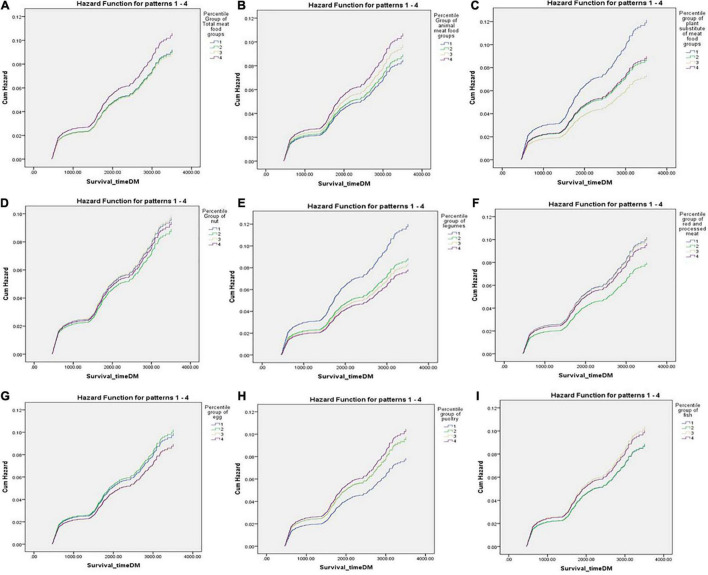
Cox proportional hazard regression plot for type 2 diabetes according to quartiles of total meats, animal sources of meat food groups, plant sources of meat food groups, nuts, legumes, red and processed meats, egg, poultry, and fish **(A–I)**.

The Cox proportional HRs for T2D according to quartiles of animal and plant sources of meat food group intakes and their subtypes are shown in Table 2. Total meat food group intake [HR: 1, 0.7 (0.6–0.9), 0.7 (0.5–0.9), 0.8 (0.6–1.06), *P* for trend = 0.01] was inversely associated with the incidence of T2D in the second model; however, our results reported no association after adjusting for confounding variables. Animal meat food groups were positively associated with T2D incidence in the second model; however, our results found no association after adjusting for confounding variables. Higher legume intake was inversely associated with the incidence of T2D [HR: 1, 0.74 (0.58–0.94), 0.69 (0.54–0.90), 0.65 (0.50–0.84), *P* for trend = 0.01)]. Dietary intakes of nuts, red and processed meats, and plant substitutes of meat food groups were not associated with incident T2D. Our finding also noted that egg intake was adversely related to incident T2D; however, this association did not change after adjusting for confounding variables. Furthermore, our results indicated that after adjusting for confounding factors, a higher fish intake [HR: 1, 1.0 (0.79–1.27), 1.17 (0.91–1.50), 1.14 (0.89–1.45)] was not associated with a higher risk of incident T2D, while the *P* for trend was significant (*P*-trend = 0.01). In subjects who reported poultry consumption of 36.4–72.8 g/day, a positive association [HR: 1.33 (1.03–1.71)] between poultry intake and T2D risk was observed after adjusting for confounding variables (Figure 2).

**TABLE 2:** Hazard ratios (HRs, 95% CI) of diabetes incidence across quartiles of animal and plant sources of meat food groups.

	Total animal and plant substitutes of meat food groups				
	Q1	Q2	Q3	Q4	P for trend
Median	61.0	96.1	134	202	
Interquartile range (g/day)	48.8–70.5	87.6–104	123–146	179–244	
Diabetes incidence (*n*)	148	130	131	140	
Model 1	1	0.9 (0.7–1.1)	0.8 (0.6–1.06)	0.8 (0.6–1.1)	0.38
Model 2	1	0.7 (0.6–0.9)	0.7 (0.5–0.9)	0.8 (0.6–1.06)	0.01
Model 3	1	0.93 (0.77–1.27)	0.86 (0.75–1.27)	1.15 (0.86–1.53)	0.30

	**Animal sources of meat food groups**				
	**Q1**	**Q2**	**Q3**	**Q4**	

Median	39.5	63.8	90.4	141	
Interquartile range (g/day)	31.1–46.4	58.0–69.8	82.7–99.2	123–178	
Diabetes incidence (*n*)	155	126	133	135	
Model 1	1	0.8 (0.6–1.0)	0.8 (0.6–1.1)	0.9 (0.7–1.1)	0.7
Model 2	1	0.9 (0.7–1.2)	1.1 (0.8–1.4)	1.2 (0.9–1.6)	0.02
Model 3	1	1.05 (0.82–1.34)	1.14 (0.89–1.42)	1.26 (0.96–1.64)	0.17

	**Plant substitute of meat food groups**				
	**Q1**	**Q2**	**Q3**	**Q4**	

Median	9.39	21.4	37.8	75.4	
Interquartile range (g/day)	6.07–12.1	18.5–24.8	32.9–43.9	62.3–96.8	
Diabetes incidence (*n*)	159	132	111	147	
Model 1	1	0.8 (0.7–1.1)	0.7 (0.6–1.0)	1.1 (0.8–1.4)	0.18
Model 2	1	0.9 (0.7–1.1)	0.8 (0.6–1.0)	1.0 (0.8–1.3)	0.36
Model 3	1	0.72 (0.56–0.92)	0.61 (0.47–0.79)	0.74 (0.57–0.97)	0.46

	**Legumes**				
	**Q1**	**Q2**	**Q3**	**Q4**	

Median	4.80	13.7	26.0	57.8	
Interquartile range (g/day)	2.74–6.83	11.1–16.6	21.1–30.4	46.1–73.9	
Diabetes incidence (n)	160	126	128	135	
Model 1	1	0.8 (0.6–1.1)	0.9 (0.73–1.1)	1.01 (0.8–1.2)	0.6
Model 2	1	0.8 (0.6–1.1)	0.9 (0.7–1.1)	0.9 (0.7–1.1)	0.9
Model 3	1	0.74 (0.58–0.94)	0.69 (0.54–0.90)	0.65 (0.50–0.84)	0.01

	**Nuts**				
	**Q1**	**Q2**	**Q3**	**Q4**	

Median	0.81	2.70	5.92	15.0	
Interquartile range (g/day)	0.43–1.33	2.28–3.39	4.85–7.45	10.7–22.2	
Diabetes incidence (*n*)	148	129	137	135	
Model 1	1	0.8 (0.6–1.0)	0.9 (0.7–1.1)	0.9 (0.7–1.1)	0.8
Model 2	1	0.9 (0.7–1.2)	0.9 (0.7–1.1)	1.0 (0.7–1.2)	0.7
Model 3	1	0.91 (0.72–1.17)	1.01 (0.79–1.28)	0.96 (0.75–1.24)	0.92

	**Red and processed meats**			
	**Q1**	**Q2**	**Q3**	**Q4**	

Median	11.8	24.12	38.2	69.6	
Interquartile range (g/day)	8.48–15.1	21.0–27.3	33.9–43.3	56.8–92.1	
Diabetes incidence (*n*)	171	123	136	119	
Model 1	1	0.8 (0.4–1.4)	0.8 (0.4–1.4)	0.5 (0.3–1.0)	0.9
Model 2	1	1.0 (0.6–1.9)	1.0 (0.6–1.9)	0.8 (0.4–1.5)	0.2
Model 3	1	0.78 (0.61–0.99)	0.98 (0.77–1.26)	0.94 (0.72–1.24)	0.83

	**Egg**			
	**Q1**	**Q2**	**Q3**	**Q4**	

Median	1.78	7.6	15.2	26.7	
Interquartile range (g/day)	1.78–3.56	7.63–7.63	15.2–15.2	22.9–38.1	
Diabetes incidence (*n*)	154	155	118	122	
Model 1	1	1.01 (0.8–1.2)	0.7 (0.6–0.9)	0.7 (0.5–0.9)	<0.001
Model 2	1	1.0 (0.8–1.3)	0.8 (0.6–1.0)	0.9 (0.7–1.2)	0.3
Model 3	1	1.03 (0.89–1.30)	0.89 (0.70–1.15)	0.90 (0.70–1.16)	0.28

	**Fish**			
	**Q1**	**Q2**	**Q3**	**Q4**	

Median	0.4	3.3	0.6	14.4	
Interquartile range (g/day)	0–0.83	1.97–3.37	4.06–6.74	12.1–20.2	
Diabetes incidence (*n*)	136	146	124	143	
Model 1	1	0.7 (0.4–1.1)	0.9 (0.6–1.4)	0.9 (0.5–1,3)	0.5
Model 2	1	0.7 (0.5–1.1)	0.9 (0.6–1.4)	0.9 (0.6–1.4)	<0.001
Model 3	1	1.0 (0.79–1.27)	1.17 (0.91–1.50)	1.14 (0.89–1.45)	0.01

	**Poultry**			
	**Q1**	**Q2**	**Q3**	**Q4**	

Median	10.7	18.2	27.1	60.7	
Interquartile range (g/day)	3.17–10.7	12.1–18.2	24.3–30.3	36.4–72.8	
Diabetes incidence (*n*)	116	149	133	151	
Model 1	1	1.3 (0.8–2.1)	1.1 (0.7–1.8)	1.4 (0.9 – 2.2)	0.7
Model 2	1	1.3 (0.8–2.0)	1.0 (0.6–1.6)	1.2 (0.8 – 1.9)	<0.001
Model 3	1	1.23 (0.96–1.98)	1.24 (0.96–1.61)	1.33 (1.03–1.71)	0.16

*The median of each quartile was utilized as a continuous variable to estimate the overall trends of HRs across quartiles of total animal- and plant-based protein food groups in the Cox proportional hazard regression models. Model 1 was crude, without adjustment; Model 2 adjusted for age and sex; Model 3 adjusted for diabetes risk score, log physical activity, education, smoking, total energy, total fat, fiber, and log sugar-sweetened beverage intakes.*

## Discussion

Our study found no significant relationship between the amount of total, animal, or plant sources of meat food group intakes with incident T2D in an adult population of the Tehran Lipid and Glucose Study. The results demonstrated that a higher intake of legumes significantly reduced the incidence of T2D. Fish (lower than one serving/day) was not associated with incident diabetes, and poultry intakes (∼two servings/day) increased the risk of T2D.

Participants who consumed higher animal sources of meat food group intakes were more likely to be current smokers than lower intakes, which was in line with previous studies (7, 18). These studies reported the positive associations of an unhealthy lifestyle in terms of smoking and high sugar-sweetened soft drinks with animal sources of meat food group intakes.

Previous studies indicate that both the quality and quantity of dietary protein determine its effect on the development of T2D (28, 29). A recent meta-analysis revealed that high intakes of both total and animal proteins are associated with increased incidence of T2D, while total and animal protein intake do not relate to T2D; nevertheless, this observation was not found for plant protein (3). Our study found no association between plant and animal sources of meat food group intake with the incidence of T2D. These results might be related to the quantity of protein intake in our study population. For example, the average percentage of protein intake in studies with a significant effect on the occurrence of T2D was ≥ 20% of energy intake in the highest group (30, 31). In contrast, the average protein intake in our study was ≥ 16% of energy intake in the highest quartile of dietary intake.

In our study, there is a big difference between the highest (46.1–73.9 g/day) and the lowest (2.74–6.83 g/day) quartile of legume intake. This food group reduced the risk of T2D following previous studies (32, 33); previous studies reported legumes as an antidiabetic food due to their low glycemic index and insoluble fiber content (34, 35). Other studies did not find a prevention role for legumes (8, 9); this controversy could be due to the different effects of various types of legumes on T2D. One cohort study had shown that consumption of lentils and chickpeas, but not dry beans or fresh peas, was related to a lower risk of T2D; however, our study did not evaluate the association of different types of legumes with T2D (32). Other pathways could describe the antidiabetic effects of legumes. First, the fiber content could alter intestine microbiota, leading to increased insulin sensitivity (36). Second, they can play a role as anti-inflammatory and antioxidant foods because legumes are high in flavonoids; these features contribute to glycemic control (37). Furthermore, legumes can take part in critical physiological processes, including glucose homeostasis due to considerable sources of minerals such as calcium, potassium, and magnesium (38).

Our study found no significant relationship observed between the amounts of nut consumption with incident T2D. In contrast, in a meta-analysis by Viguiliouk et al. nuts at a median dose of 56 g/day significantly lowered HbA1c and fasting glucose compared with control diets (39). Several factors might be responsible for our study’s apparent lack of effect of nuts on T2D. The maximum daily consumption of nuts in the fourth quartile is 15 g per day, which is almost a quarter of the median consumption of nuts in the study of Viguiliouk et al. We did not have data on the type of nuts consumed. Each type of nut, such as almonds, walnuts, hazelnuts, cashews, pistachios, etc., has different effects on metabolic profiles. For example, almonds effectively lower the glycemic index (40), while walnut consumption has reduced sensitivity to lipid peroxidation by increasing total plasma antioxidant capacity (41).

In our study, egg consumption could not change the risk of developing T2D; however, results from other studies are inconsistent with our findings; in a cohort study, consuming more eggs (more than 45 g per day) was associated with a 45% reduction in the risk of T2D (42). Also, different results may be due to differences in dietary habits related to egg consumption, which could affect the incidence of T2D. For example, in western countries, eggs are commonly eaten with processed meats such as sausage, bacon, or burgers, which are themselves associated with a higher risk of T2D (7), while in Korea, boiled or baked eggs are often consumed in isolation, not as part of a mixed dish.

Based on our results, poultry could increase T2D incidence; previous studies do not support a clear overall association of poultry intake with diabetes risk (18, 19). In Iran, high-heat cooking methods such as broiling, barbecuing/grilling, and roasting grilling are mostly used to prepare chicken and fish. During high-temperature cooking, the production of hazardous chemicals, including heterocyclic aromatic amines, polycyclic aromatic hydrocarbons, and advanced glycation end products induce gene expression changes in JAK/STAT and MAPK pathways that are linked with inflammation and diabetes (43, 44).

One study reported that fish [RR = 0.89 (CI: 0.81–0.98), per 100 g/day] and marine omega-3 fatty acid [RR = 0.87 (CI: 0.79–0.96)] intake was inversely associated with the risk of diabetes in a subgroup analysis of five Asian cohorts (45). In contrast, the fish intake of our study population was much lower than 100 g/day. Another study in North America and Europe reported a positive association between fish intake and risk of diabetes which may be due to environmental contaminants in fish such as dioxins and methyl mercury which may interfere with insulin signaling pathways (17, 18, 46).

Moreover, in previous observational studies, fish and omega-3 fatty acid intakes were heterogeneously related to glucose tolerance. Omega-3 fatty acids may raise glucose concentration through lowering glucose utilization and increasing glucagon-stimulated C-peptide or increasing hepatic gluconeogenesis through high uptake and oxidation of free fatty acids in the liver, which induce lowering TG. Therefore, fish and omega-3 fatty acid consumption may raise the incidence of T2D by increasing the serum concentration of glucose. This pathway does not cause other adverse metabolic abnormalities, such as insulin resistance, high TG, and low HDL-C (46).

Our results suggested no relationship between red and processed meat intake and the incidence of T2D in the Tehranian population. In the western population, red and processed meat consumption has been related to higher risks of several chronic diseases, including diabetes. In contrast, each 100 g/day increase in red meat consumption was associated with a 13 and 19% higher risk of diabetes in two large meta-analyses of prospective cohort studies (7, 19). In the Asian population, the meat consumption pattern is different (18). A previous study in this population found no significant relationship between red meat intake and the incidence of metabolic syndrome (47). This discrepancy in the relationship of red meat intake with diabetes may be due to substantially lower consumption of red meat in our study population (total red meat intake in the highest quartile was approximately one serving/day) and in the Asian population compared with the Western.

Our research had some strengths to point out. First, the population-based TLGS has well-founded data that display the urban population of Tehran. Second, the definition of diabetes was based on fasting blood experiments. Finally, we assessed all aspects of dietary factors, including fiber and fatty acids, on the occurrence of diabetes and eliminated probable confounders in our final analysis.

Our study had several limitations to announce. First, we had no data about common cooking ways in some primary animal-based protein sources, so we could not measure this effect on the development of T2D. Second, although we tried to identify all confounding variables in our adjusted methods, some residual confounding might not be omitted due to a lack of knowledge or measurements. Finally, insulin sensitivity, as a sensitive marker, was not measured, so we could not detect the association between protein food group consumption and the risk of insulin resistance. Considering dietary intake at a one-time point is one of the limitations of this study, so we do not examine changes in dietary habits during follow-up years. Expert dietitians collect information on dietary intakes without food replicas. A previous systematic review reported significant under-reporting of energy intake at the group levels (ranging from 4.6 to 42%) in all studies using an FFQ compared with the doubly labeled water method (48). The percentage of misreporting in our research fell within this range.

## Conclusion

In conclusion, our findings revealed that both plant and animal sources of meat food group intakes did not significantly associate with the risk of new-onset T2D. In addition, a diet rich in legumes significantly reduced the risk of T2D incidence. A diet high in poultry increased the risk of T2D incidence, probably due to high-temperature cooking methods and environmental contaminants. Also, there was no significant and consistent association between red and processed meats and diabetes risk due to substantially lower consumption of red meat in our study population compared with the Western countries.

## Data Availability Statement

The raw data supporting the conclusions of this article will be made available by the authors, without undue reservation.

## Ethics Statement

The studies involving human participants were reviewed and approved by Research Institute for Endocrine Sciences, Shahid Beheshti University of Medical Sciences, Tehran, Iran. The patients/participants provided their written informed consent to participate in this study.

## Author Contributions

FH-E and GK: conceptualization. FH-E and NB: formal analysis. FH-E, GK, and NB: methodology. FA and PM: supervision and writing – review and editing. GK, NB, and FH-E: writing – original draft. All authors contributed to the article and approved the submitted version.

## Conflict of Interest

The authors declare that the research was conducted in the absence of any commercial or financial relationships that could be construed as a potential conflict of interest.

## Publisher’s Note

All claims expressed in this article are solely those of the authors and do not necessarily represent those of their affiliated organizations, or those of the publisher, the editors and the reviewers. Any product that may be evaluated in this article, or claim that may be made by its manufacturer, is not guaranteed or endorsed by the publisher.
